# Supplementary material for: Peanut oil is more environmentally sustainable than rapeseed oil from a carbon and nitrogen footprint perspective in China
Source: PeerJ. 2025 Aug 28;13:e19941. doi: 10.7717/peerj.19941 (PMC12399079; doi:10.7717/peerj.19941)
Supplement: Supplemental Information 2 [file peerj-13-19941-s002.docx]

**Supplementary Information**


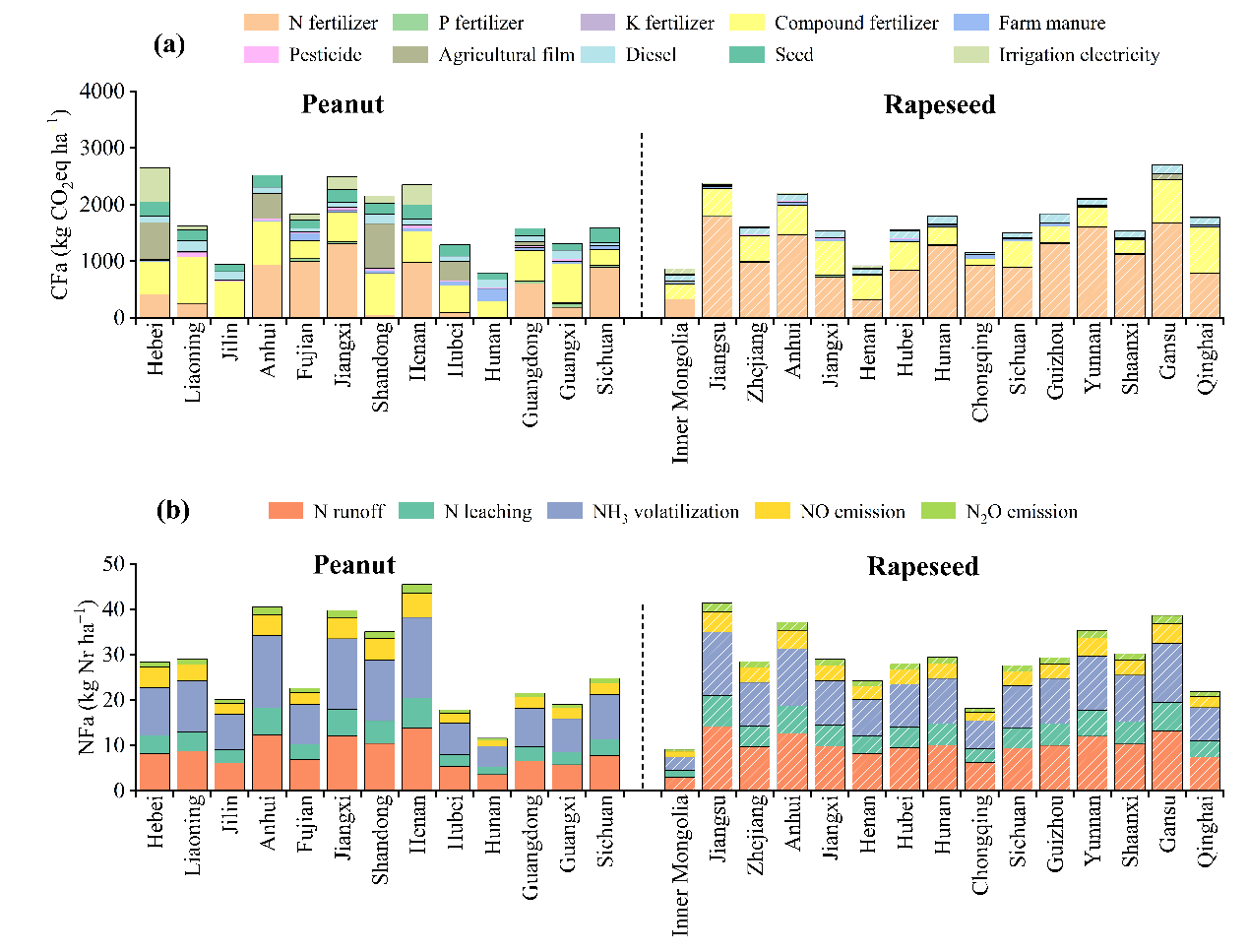


**Figure S1.** The compositions of the CF and NF in planting phase for peanut and rapeseed in main producing provinces during 2004−2023.

**Table S1** Specific geographic information of the study area.

| Province | Latitude (N) | Longitude (E) | Altitude (m) |
| --- | --- | --- | --- |
| **Major peanut-producing regions** | | | |
| Hebei | 39.41 | 116.66 | 509 |
| Liaoning | 41.09 | 122.31 | 224 |
| Jilin | 43.64 | 126.47 | 404 |
| Anhui | 32.06 | 117.26 | 195 |
| Fujian | 25.96 | 118.29 | 482 |
| Jiangxi | 27.32 | 116.03 | 246 |
| Shandong | 36.42 | 118.75 | 90 |
| Henan | 33.92 | 113.50 | 243 |
| Hubei | 31.18 | 112.25 | 431 |
| Hunan | 27.42 | 111.52 | 357 |
| Guangdong | 22.89 | 113.48 | 218 |
| Guangxi | 23.67 | 108.27 | 385 |
| Sichuan | 30.27 | 102.95 | 2560 |
| **Major rapeseed-producing regions** | | | |
| Inner Mongolia | 45.94 | 111.62 | 999 |
| Jiangsu | 32.98 | 119.16 | 15 |
| Zhejiang | 29.18 | 120.59 | 303 |
| Anhui | 32.06 | 117.26 | 195 |
| Jiangxi | 27.32 | 116.03 | 246 |
| Henan | 33.92 | 113.50 | 243 |
| Hubei | 31.18 | 112.25 | 431 |
| Hunan | 27.42 | 111.52 | 357 |
| Chongqing | 30.21 | 107.74 | 730 |
| Sichuan | 30.27 | 102.95 | 2560 |
| Guizhou | 26.94 | 106.60 | 1108 |
| Yunnan | 25.27 | 101.86 | 1892 |
| Shaanxi | 37.73 | 112.39 | 1130 |
| Gansu | 37.87 | 100.52 | 2158 |
| Qinghai | 35.50 | 96.24 | 4049 |

Geographic information sources: National Platform for Common GeoSpatial Information Services (https://www.tianditu.gov.cn/).

**Table S2** Carbon emission factors of agricultural inputs used for CF calculation.

| Agricultural inputs | Emission factors | Reference |
| --- | --- | --- |
| Urea | 2.041 kg CO_2_eq kg^−1^ | (Chen et al. 2015) |
| Ammonium bicarbonate | 1.928 kg CO_2_eq kg^−1^ | (Chen et al. 2015) |
| Other N fertilizer | 2.116 kg CO_2_eq kg^−1^ | (Chen et al. 2015) |
| Calcium superphosphate | 0.195 kg CO_2_eq kg^−1^ | (Chen et al. 2015) |
| Diammonium phosphate | 1.109 kg CO_2_eq kg^−1^ | (Chen et al. 2015) |
| Other P fertilizer | 0.636 kg CO_2_eq kg^−1^ | (Chen et al. 2015) |
| Potassium chloride | 0.168 kg CO_2_eq kg^−1^ | (Chen et al. 2015) |
| Other K fertilizer | 0.180 kg CO_2_eq kg^−1^ | (Chen et al. 2015) |
| Compound fertilizer | 1.77 kg CO_2_eq kg^−1^ | (Huang et al. 2017) |
| Pesticides | 14.319 kg CO_2_eq kg^−1^ | (Zhang et al. 2016) |
| Agri-film | 22.70 kg CO_2_eq kg^−1^ | (Huang et al. 2017) |
| Diesel production | 0.89 kg CO_2_eq L^−1^ | (Huang et al. 2017) |
| Diesel combustion | 4.10 kg CO_2_eq L^−1^ | (Chen et al. 2020) |
| Peanut seed | 0.92 kg CO_2_eq kg^−1^ | (West and Marland 2002) |
| Rapeseed seed | 0.83 kg CO_2_eq kg^−1^ | (Gan et al. 2012) |

**Table S3** Carbon emission factors of irrigation electricity consumption in different regions of China used for CF calculation.

| Regions | Carbon emission factors  (kg CO_2_eq kWh^−1^) | Reference |
| --- | --- | --- |
| Hebei | 0.7252 | https://www.mee.gov.cn/xxgk2018/xxgk/xxgk01/202412/t20241226_1099413.html |
| Liaoning | 0.5626 |  |
| Jilin | 0.4932 |  |
| Anhui | 0.6782 |  |
| Fujian | 0.4092 |  |
| Jiangxi | 0.5752 |  |
| Shandong | 0.6410 |  |
| Henan | 0.6058 |  |
| Hubei | 0.4364 |  |
| Hunan | 0.4900 |  |
| Guangdong | 0.4403 |  |
| Guangxi | 0.4044 |  |
| Sichuan | 0.1404 |  |
| Inner Mongolia | 0.6849 |  |
| Jiangsu | 0.5978 |  |
| Zhejiang | 0.5153 |  |
| Chongqing | 0.5227 |  |
| Guizhou | 0.4989 |  |
| Yunnan | 0.1073 |  |
| Shaanxi | 0.6558 |  |
| Gansu | 0.4772 |  |
| Qinghai | 0.1567 |  |
| Average | 0.5366 |  |

**Table S4** Nr emission factors of agricultural inputs used for NF calculation.

| Agricultural inputs | N_2_O emission  (g N kg^−1^) | NO emission (g N kg^−1^) | NH_3_ volatilization  (g N kg^−1^) | Reference |
| --- | --- | --- | --- | --- |
| N fertilizer | 0.110 | 16.520 | 2.256 | (Liang 2009) |
| P fertilizer | 0.011 | 2.155 |  | (Liang 2009) |
| K fertilizer | 0.017 | 2.897 |  | (Liang 2009) |
| Diesel production | 0.077 | 0.835 |  | (Liang 2009) |
| Diesel combustion | 0.010 | 2.237 |  | (Liang 2009) |
| Agricultural film | 0.19 | 15.1 |  | (Xia et al. 2016) |
| Insecticide | 0.1661 | 13.18 |  | (Chen et al. 2019) |
| Herbicide | 0.1015 | 8.06 |  | (Chen et al. 2019) |
| Fungicide | 0.1057 | 8.41 |  | (Chen et al. 2019) |

**Table S5** Direct N_2_O emission factors in different regions of China used for CF calculation.

| Regions | Direct N_2_O emission factors (%) | Reference |
| --- | --- | --- |
| Inner Mongolia, Gansu, Qinghai, Shaanxi | 0.56 | (NDRC 2011) |
| Jilin, Liaoning | 1.14 |  |
| Hebei, Henan, Shandong | 0.57 |  |
| Zhejiang, Jiangsu, Anhui, Jiangxi, Hubei, Hunan, Chongqing, Sichuan | 1.09 |  |
| Guangdong, Guangxi, Fujian | 1.78 |  |
| Guizhou, Yunnan | 1.06 |  |

**Table S6** Emission factors of reactive N from N fertilization in the field used for NF calculation.

| Nr forms | Emission factors (kg N kg^−1^) | | Reference |
| --- | --- | --- | --- |
| N_2_O emission | 0.01 | | (IPCC 2019) |
| NO emission | 0.00392 | | (Li et al. 2022) |
| NH_3_ volatilization | Peanut | 0.091652 | (Li et al. 2022) |
|  | Rapeseed | 0.070284 | (Li et al. 2022) |
| N leaching | 0.035 | | (Xia et al. 2018) |
| N runoff | 0.073 | | (Xia et al. 2018) |

**References**

**Chen S, Lu F, and Wang XK. 2015**. Estimation of greenhouse gases emission factors for China's nitrogen，phosphate, and potash fertilizers. *Acta Ecologica Sinica* **35**:6371-6383.

**Chen ZD, Xu CC, Ji L, and Fang FP. 2019**. Comprehensive evaluation for carbon and nitrogen footprints of rice–wheat rotation system in Middle Yangtze River Basin. *Journal of Plant Nutrition and Fertilizers* **25**:1125–1133.

**Chen ZD, Xu CC, Ji L, Feng JF, Li FB, Zhou XY, and Fang FP. 2020**. Effects of multi-cropping system on temporal and spatial distribution of carbon and nitrogen footprint of major crops in China. *Global Ecology and Conservation* **22**:e00895.

**NDRC. 2011**. *Guidelines for the Preparation of Provincial Greenhouse Gas Inventories*. National Development and Reform Commission (NDRC), Beijing, China.

**Gan YT, Liang C, Huang GB, Malhi SS, and Katepa-Mupondwa F. 2012**. Carbon footprint of canola and mustard is a function of the rate of N fertilizer. *The International Journal of Life Cycle Assessment* **17**:58-68.

**Huang XM, Chen CQ, Qian HY, Chen MZ, Deng AX, Zhang J, and Zhang WJ. 2017**. Quantification for carbon footprint of agricultural inputs of grains cultivation in China since 1978. *Journal of Cleaner Production* **142**:1629-1637.

**IPCC. 2019**. Guidelines for National Greenhouse Gas Inventories. National Greenhouse Gas Inventories Programme, Intergovernmental Panel on Climate Change: Hayama, Japan.

**Li YP, Wu WA, Yang JX, Cheng K, Smith P, Sun JF, Xu XR, Yue Q, and Pan GX. 2022**. Exploring the environmental impact of crop production in China using a comprehensive footprint approach. *Science of the Total Environment* **824**:824.

**Liang L. 2009**. Discussion and empirical study on environmental impact Assessment method of circular agriculture based on LCA Doctoral Dissertation. China Agricultural University.

**West TO, and Marland G. 2002**. A synthesis of carbon sequestration, carbon emissions, and net carbon flux in agriculture: comparing tillage practices in the United States. *Agriculture Ecosystems & Environment* **91**:217-232.

**Xia LL, Ti CP, Li BL, Xia YQ, and Yan XY. 2016**. Greenhouse gas emissions and reactive nitrogen releases during the life-cycles of staple food production in China and their mitigation potential. *Science of The Total Environment* **556**:116-125.

**Xia YQ, Yang WX, Shi WM, and Yan XY. 2018**. Estimation of non-point source N emission in intensive cropland of China. *Journal of Ecology and Rural Environment* **34**:782-787.

**Zhang G, Lu F, Huang ZG, Chen S, and Wang XK. 2016**. Estimations of application dosage and greenhouse gas emission of chemical pesticides in staple crops in China. *Chinese Journal of Applied Ecology* **27**:2875-2883.
